# Prognostic significance of preoperative plasma D-dimer level in patients with surgically resected clinical stage I non-small cell lung cancer: a retrospective cohort study

**DOI:** 10.1186/s13019-017-0676-3

**Published:** 2017-11-28

**Authors:** Kaoru Kaseda, Keisuke Asakura, Akio Kazama, Yukihiko Ozawa

**Affiliations:** 1 Department of Thoracic Surgery, Sagamihara Kyodo Hospital, 2-8-18 Hashimoto, Midori-ku, Sagamihara, Kanagawa 252-5188 Japan; 2 Department of Pathology, Sagamihara Kyodo Hospital, 2-8-18 Hashimoto, Midori-ku, Sagamihara, Kanagawa 252-5188 Japan; 3 Yuai Clinic, 1-6-2 Kitashinyokohama, Kohoku-Ku, Yokohama, Kanagawa 223-0059 Japan

**Keywords:** D-dimer, Non-small cell lung cancer, Prognosis

## Abstract

**Background:**

Plasma D-dimer level, a marker of hypercoagulation, has been reported to be associated with survival in several types of cancers. The present study aimed to evaluate the prognostic significance of preoperative D-dimer levels in patients with surgically resected clinical stage I non-small cell lung cancer (NSCLC).

**Methods:**

Participants comprised 237 patients with surgically resected clinical stage I NSCLC. In addition to factors such as age, sex, and smoking status, the association between preoperative D-dimer level and survival was explored.

**Results:**

Patients were divided into two groups according to D-dimer level: Group A, ≤ 1.0 μg/ml (*n* = 170); and Group B, > 1.0 μg/ml (*n* = 67). The 5-year recurrence-free survival rate was 81.6% for Group A and 66.6% for Group B (*p* < 0.001). The 5-year overall survival rate was 93.6% for Group A and 84.7% for Group B (*p* = 0.002). Multivariate survival analysis identified D-dimer level as an independent prognostic factor, along with age, maximum standardized uptake value of the primary tumor, and pathological stage.

**Conclusions:**

Preoperative D-dimer level is an independent prognostic factor in patients with surgically resected clinical stage I NSCLC.

## Background

Patients with malignant tumors sometimes develop hypercoagulability, which can present as conditions like disseminated intravascular coagulation (DIC) and venous thromboembolism (VTE). Systemic hypercoagulability is frequently observed in patients with advanced-stage cancer, even if no thrombosis is present. One previous study expanded the definition of Trousseau’s syndrome to include chronic disseminated intravascular coagulopathy associated with microangiopathy, verrucous endocarditis and arterial emboli in patients with cancer, and this syndrome is frequent among patients with mucin-positive carcinoma [1]. More recently, the term has been applied to a wide variety of clinical situations, from classic descriptions to any form of coagulopathy occurring against a background of malignancy [2].

Physiological degradation of fibrin results in plasma D-dimer (D-dimer), and levels of this stable end product are increased by significant fibrin formation and fibrinolysis. D-dimer level thus offers an indicator of the hypercoagulable state often evident in patients with thrombosis or DIC. As levels of D-dimer are increased following clotting, measurement of D-dimer concentration is a standard initial assessment for suspected cases of acute VTE [3]. Increased concentrations of D-dimer are also seen with other situations, including infection, pregnancy, and cancer, and after trauma or surgery.

Tumor-induced coagulation and fibrin formation have been reported as prerequisite to tumor angiogenesis, metastasis and invasion, with cross-linked fibrin in the extracellular matrix providing the basis for endothelial cell and tumor cell migration during angiogenesis and invasion [4, 5]. Various investigations have reported that D-dimer levels, reflecting the degree of coagulation and fibrinolytic activation, correlate with tumor stage, response to chemotherapy and prognosis for several types of cancer [6–9], including non-small cell lung cancer (NSCLC) [9–11]. However, the relevance of D-dimer level for primary lung cancer has yet to be established. This study aimed to elucidate the prognostic significance of preoperative D-dimer levels after complete resection of clinical stage I NSCLC.

## Methods

### Patient eligibility

Between April 2007 and August 2013, a total of 306 consecutive patients with potentially resectable NSCLC underwent measurement of D-dimer levels at Sagamihara Kyodo Hospital, Kanagawa, Japan. D-dimer level was measured within 4 weeks of surgery. We reviewed the data from 237 of these patients who were diagnosed with clinical stage I NSCLC according to the seventh edition of the TNM Staging Classification for Lung Cancer [12]. Patients who underwent incomplete resection or neoadjuvant chemotherapy/radiotherapy were excluded.

In addition to the D-dimer level, we reviewed the medical records of each patient for the following clinicopathological information: age, sex, smoking habit, serum concentration of carcinoembryonic antigen (CEA), extent of pulmonary resection, tumor location, maximum standardized uptake value (SUVmax) of the primary tumor, maximum tumor diameter, histological type, grade, pleural invasion, and pathological stage. All clinical, intraoperative, radiological, and pathological findings from two hospitals in Kanagawa, Japan (Sagamihara Kyodo Hospital and Yuai Clinic) were reviewed. Histological classification of NSCLC was based on the criteria of the World Health Organization [13]. Pre- and postoperative staging was based on the TNM staging system. Data collection and analyses were approved, and the need to obtain written informed consent from each patient was waived by the first author’s institutional review board, due to the retrospective nature of this investigation.

### Computed tomography

Diagnostic-quality contrast-enhanced computed tomography (CT) of the chest with a slice thickness of 5 mm was performed for all patients. A tumor was deemed central if the center was located in the inner one-third of the lung parenchyma (adjacent to the mediastinum) on transverse CT. Peripherally located tumors were identified as those centered in the outer two-thirds of the lung parenchyma on transverse CT. The maximal diameter of lung nodules was measured on contrast-enhanced chest CT. All imaging was performed within 4 weeks of surgery.

### Integrated ^18^F–fluorodeoxyglucose positron emission tomography imaging

Each patient underwent integrated ^18^F–fluorodeoxyglucose positron emission tomography/CT (FDG-PET/CT) imaging before surgical resection. All integrated FDG-PET/CT imaging was performed within 4 weeks of surgery. After fasting for 6 h, FDG (3.5 MBq/kg body weight) was intravenously injected if the blood sugar level was lower than 200 mg/dl. Image acquisition was started 60 min after the injection using a single PET/CT combined scanner (Eminence-SOPHIA; Shimadzu, Kyoto, Japan) [14]. Image emission data from the eyes to the mid-thigh area were continuously acquired over a period of approximately 20 min. After attenuation corrections were made for the resulting image data, reconstruction was performed using a dynamic row-action expectation maximization algorithm [15]. The reconstructed sectional images were then evaluated both visually and quantitatively using the SUV_max_ inside a volume of interest (VOI) placed on the lesions. SUV_max_ was calculated as follows: [(maximum activity in VOI) ⁄ (volume of VOI)] ⁄ [(injected FDG dose) ⁄ (patient weight)]. The quality of radiation measurements of the PET/CT scanner was assured by calibration in accordance with National Electrical Manufacturers Association NU-2 2001 standards [16].

Nodal uptake with an SUV_max_ > 2.5 was considered positive. To determine the SUV, a cylindrical region of interest (ROI) was placed over the tumor site manually on the hottest trans-axial slice. Activity within the ROI was determined and expressed as the SUV, defined as the ratio of activity in the tissue to the decay-corrected activity injected into the patient. All SUV measurements were normalized for patient body weight. SUV_max_ within an ROI was used as the reference measurement.

Three experienced radiologists individually analyzed the integrated FDG-PET/CT images, with the final assessment made by consensus if initial assessments differed.

### Surgical resection

All patients underwent anatomical lung resection (lobectomy or greater and segmentectomy) and radical lymphadenectomy in our hospital. All surgical resections were performed by thoracic surgeons at Sagamihara Kyodo Hospital. All surgical resection techniques were standardized. Systematic lymph node dissection was performed in all patients according to the criteria of the American Thoracic Society, removing at least three hilar and three mediastinal stations.

### Pathological examination

All resected tumor specimens were examined by experienced pulmonary pathologists. Histological classification of NSCLC was based on WHO classifications. Dissected lymph nodes were histologically examined following hematoxylin and eosin staining.

### Statistical analysis

Statistical analysis was performed using SPSS version 23.0 software (IBM Corporation, Armonk, NY). Survival curves were constructed using the Kaplan-Meier method. Recurrence-free survival (RFS) probabilities and overall survival (OS) rates were compared using the log-rank test. The Cox proportional hazard model was used to estimate hazard ratio (HR) with 95% confidence interval (CI) for uni- and multivariate analyses. All tests were two-sided, and values of *P* < 0.05 were considered statistically significant. Factors identified as significant in univariate analysis (*P* < 0.05) were entered into the multivariate analysis.

## Results

### Patient characteristics

Patients were divided into two groups according to the D-dimer level: Group A, ≤ 1.0 μg/ml (*n* = 170); and Group B, > 1.0 μg/ml (*n* = 67). Patient characteristics are shown in Table 1. Participants comprised 140 men and 97 women, ranging in age from 31 to 85 years (median, 69 years). Median observation period in the survivors was 60.0 months (range, 2–110 months). Associations between D-dimer level and clinicopathological characteristics are shown in Table 2. D-dimer level correlated with serum CEA (*p* < 0.001), SUVmax of the primary tumor (*p* < 0.001), tumor size (*p* = 0.023), and pathological stage (*p* = 0.003).

**Table 1:** Clinicopathological characteristics of 237 patients with clinical stage I NSCLC

Variables	n (%) or mean ± SD
Age at operation, y	69.0 ± 9.7
Gender	
Female	97 (40.9%)
Male	140 (59.1%)
Smoking habit	
Never smoker	94 (39.7%)
Ever smoker	143 (60.3%)
Serum CEA, ng/ml	
≤ 5	178 (75.1%)
> 5	59 (24.9%)
Extent of pulmonary resection	
Segmentectomy	34 (14.3%)
Lobectomy or more	203 (85.7%)
Tumor location	
Central	24 (10.1%)
Non-central	213 (89.9%)
SUV_max_ of primary tumor	2.3 ± 2.8
D-dimer, μg/ml	
≤ 1.0	170 (71.7%)
> 1.0	67 (28.3%)
Tumor size, cm	2.7 ± 1.5
Histological type	
AD	188 (79.3%)
SQ	36 (15.3%)
Others	13 (5.4%)
Grade	
1	156 (65.8%)
2 / 3 / 4	81 (34.2%)
Pleural invasion	
Absent	209 (88.2%)
Present	28 (11.8%)
Pathological stage	
Stage I	205 (86.4%)
Stage II	19 (8.2%)
Stage III	13 (5.4%)

*NSCLC* non-small cell lung cancer, *SD* standard deviation, *CEA* carcinoembryonic antigen, *AD* adenocarcinoma, *SQ* squamous cell carcinoma, *SUV*_*max*_ maximum standardized uptake value

**Table 2:** Association between D-dimer level and clinicopathological characteristics in patients with clinical stage I NSCLC

Variables	GroupA:D-dimer, μg/ml ≤1.0	GroupB:D-dimer, μg/ml >1.0	*P* value
	(*n* = 170)	(*n* = 67)	
	n (%)	n (%)	
Age at operation, y			
< 70	82 (48.1%)	31 (46.3%)	0.785
≥ 70	88 (51.9%)	36 (53.7%)	
Gender			
Female	68 (40.0%)	29 (43.3%)	0.643
Male	102 (60.0%)	38 (56.7%)	
Smoking habit			
Never smoker	70 (41.2%)	24 (35.8%)	0.448
Ever smoker	100 (58.8%)	43 (64.2%)	
Serum CEA, ng/ml			
≤ 5	140 (82.3%)	38 (56.7%)	<0.001
> 5	30 (17.7%)	29 (43.3%)	
Extent of pulmonary resection			
Segmentectomy	24 (14.1%)	10 (14.9%)	0.873
Lobectomy or more	146 (85.9%)	57 (85.1%)	
Tumor location			
Central	16 (9.4%)	8 (12.0%)	0.561
Non-central	154 (90.6%)	59 (88.0%)	
SUV_max_ of primary tumor			
≤ 2.3	123 (72.4%)	28 (41.8%)	<0.001
> 2.3	47 (27.6%)	39 (58.2%)	
Tumor size, cm			
≤ 3	118 (69.4%)	36 (53.7%)	0.023
> 3	52 (30.6%)	31 (46.3%)	
Histological type			
AD	139 (81.7%)	49 (73.1%)	0.140
Others	31 (28.3%)	18 (26.9%)	
Grade			
1	113 (72.4%)	43 (64.2%)	0.738
2 / 3 / 4	57 (27.6%)	24 (35.8%)	
Pleural invasion			
Absent	154 (90.6%)	55 (82.0%)	0.068
Present	16 (9.4%)	12 (18.0%)	
Pathological stage			
Stage I	154 (90.6%)	51 (76.1%)	0.003
Stage II / III	16 (9.4%)	16 (23.9%)	

*NSCLC* non-small cell lung cancer, *CEA* carcinoembryonic antigen, *AD* adenocarcinoma, *SUVmax* maximum standardized uptake value

### Survival analysis of patients with pathological stage I adenocarcinoma after surgical resection

**Fig. 1:**
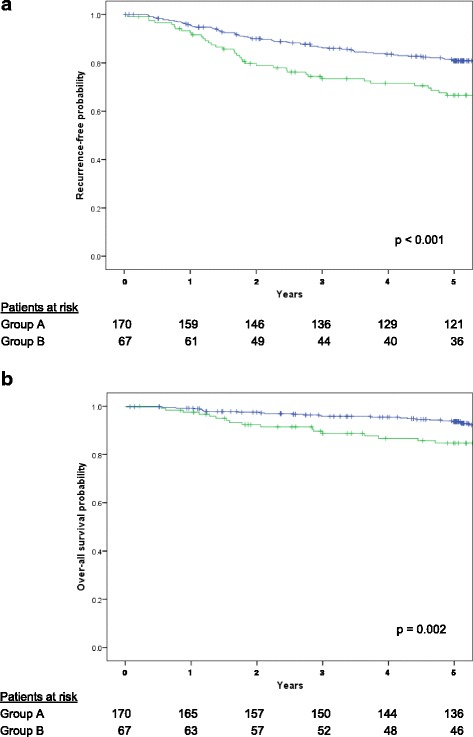
**a** Recurrence-free survival of patients with surgically resected clinical stage I non-small cell lung cancer. The 5-year recurrence-free survival rate was 81.6% for Group A, and 66.6% for Group B (*p* < 0.001). **b** Overall survival of patients with surgically resected clinical stage I non-small cell lung cancer. The 5-year overall survival rate was 93.6% for Group A, and 84.7% for Group B (*p* = 0.002)

The 5-year RFS rate was 81.6% for Group A, and 66.6% for Group B (p < 0.001) (Fig. 1a). The 5-year OS rate was 93.6% for Group A, and 84.7% for Group B (*p* = 0.002) (Fig. 1b).

Univariate survival analysis revealed age (<70 vs. ≥70, OS: HR 2.31; 95%CI 1.12–5.13), serum CEA (≤5 vs. >5, RFS: HR 2.11; 95%CI 1.03–4.12, OS: HR 2.42; 95%CI 1.31–5.46), SUVmax of the primary tumor (≤2.3 vs. >2.3, RFS: HR 5.89; 95%CI 3.45–14.2, OS: HR 3.89; 95%CI 1.64–9.01), D-dimer level (≤1.0 vs. >1.0, RFS: HR 2.62; 95%CI 1.41–5.61, OS: HR 2.67; 95%CI 1.23–5.12), pleural invasion (absent vs. present, OS: HR 2.42; 95%CI 1.06–5.52) and pathological stage (Stage I vs. Stage II/III, RFS: HR 5.61; 95%CI 3.22–9.12, OS: HR 3.91; 95%CI 1.65–8.98) as significant prognostic factors (Table 3). Multivariate survival analysis identified age (<70 vs. ≥70, OS: HR 2.39; 95%CI 1.09–5.24), SUVmax of the primary tumor (≤2.3 vs. >2.3, RFS: HR 3.78; 95%CI 1.61–8.72, OS: HR 3.43; 95%CI 1.36–8.61), D-dimer level (≤1.0 vs. >1.0, RFS: HR 1.92; 95%CI 1.33–2.91, OS: HR 2.24; 95%CI 1.05–4.69), and pathological stage (Stage I vs. Stage II/III, RFS: HR 4.01; 95%CI 1.64–9.91, OS: HR 4.11; 95%CI 1.65–9.98) as significant prognostic factors (Table 4).

**Table 3:** Univariate analyses for recurrence-free and overall survival in patients with clinical stage I NSCLC

	RFS		OS	
Variables	HR (95% CI)	*P* value	HR (95% CI)	*P* value
Age at operation, y				
<70	1		1	
≥70	1.12 (0.56–2.14)	0.762	2.31 (1.12–5.13)	0.031
Gender				
Female	1		1	
Male	1.13 (0.54–2.28)	0.668	1.02 (0.78–1.52)	0.869
Smoking habit				
Never smoker	1		1	
Ever smoker	1.22 (0.88–1.72)	0.281	1.21 (0.86–1.82)	0.225
Serum CEA, ng/ml				
≤5	1		1	
>5	2.11 (1.03–4.12)	0.049	2.42 (1.31–5.46)	0.019
Extent of pulmonary resection				
Segmentectomy	1		1	
Lobectomy or more	0.75 (0.56–1.21)	0.221	0.81 (0.55–1.26)	0.231
Tumor location				
Central	1		1	
Non-central	0.94 (0.44–1.86)	0.852	0.58 (0.79–4.22)	0.586
SUV_max_ of primary tumor				
≤2.3	1		1	
>2.3	5.89 (3.45–14.2)	<0.001	3.89 (1.64–9.01)	0.001
D-dimer, μg/ml				
≤1.0	1		1	
>1.0	2.62 (1.41–5.61)	0.019	2.67 (1.23–5.12)	0.021
Tumor size, cm				
≤3	1		1	
>3	1.32 (0.64–2.72)	0.483	1.16 (0.54–2.76)	0.688
Histological type				
AD	1		1	
Others	1.33 (0.64–2.76)	0.486	1.35 (0.64–2.81)	0.479
Grade				
1	1		1	
2 / 3 / 4	1.33 (0.61–2.79)	0.438	1.41 (0.68–2.82)	0.439
Pleural invasion				
Absent	1		1	
Present	2.42 (1.06–5.52)	0.038	1.95 (0.76–5.01)	0.181
Pathological stage				
Stage I	1		1	
Stage II/III	5.61 (3.22–9.12)	<0.001	3.91 (1.65–8.98)	0.001

*NSCLC* non-small cell lung cancer, *CEA* carcinoembryonic antigen, *AD* adenocarcinoma, *SUVmax* maximum standardized uptake value, *RFS* recurrence-free survival, *OS* overall survival, *HR* hazard ratio, *CI* confidence interval

**Table 4:** Multivariate analyses for recurrence-free and overall survival in patients with clinical stage I NSCLC

	RFS		OS	
Variables	HR (95% CI)	*P* value	HR (95% CI)	*P* value
Age at operation, y				
< 70			1	
≥ 70	–	–	2.39 (1.09–5.24)	0.029
Serum CEA, ng/ml				
≤ 5	1		1	
> 5	1.03 (0.98–1.05)	0.061	1.74 (0.79–3.81)	0.163
SUV_max_ of primary tumor				
≤ 2.3	1		1	
> 2.3	3.78 (1.61–8.72)	0.002	3.43 (1.36–8.61)	0.008
D-dimer, μg/ml				
≤ 1.0	1		1	
> 1.0	1.92 (1.33–2.91)	0.041	2.24 (1.05–4.69)	0.032
Pleural invasion				
Absent	1			
Present	1.05 (0.98–1.09)	0.071	–	–
Pathological stage				
Stage I	1		1	
Stage II/III	4.01 (1.64–9.91)	0.003	4.11 (1.65–9.98)	0.003

*NSCLC* non-small cell lung cancer, *CEA* carcinoembryonic antigen, *SUV*_*max*_ maximum standardized uptake value, *RFS* recurrence-free survival, *OS* overall survival, *HR* hazard ratio, *CI* confidence interval

## Discussion

This retrospective investigation examined the prognostic significance of preoperative D-dimer concentrations in patients after surgical resection of clinical stage I NSCLC. Factors affecting the prognosis of surgically resected NSCLC have not yet been characterized in detail. However, clinicopathological factors such as positive cytological findings from pleural lavage, high preoperative concentrations of CEA, high tumor SUVmax and presence of lymphovascular invasion have been reported as associated with recurrence or decreased survival after surgery for NSCLC [17–19]. As a product of fibrin degradation, D-dimer is produced when cross-linked fibrin is broken down by plasmin-induced fibrinolysis. Concentrations of D-dimer are considered to represent a global biomarker of hemostasis and fibrinolysis. The processes of metastasis and tumor growth involve various interactions between the tumor and host. Metastatic cancer cells must separate from the primary tumor, enter the circulation, attach to the vasculature of the destination, invade the tissue at this new site and establish neovasculature [20, 21]. Following initial cancer cell arrest in the vasculature of the destination organ, clotted plasma and platelets act in concert to stabilize circulating cancer cells by generating a thrombus that facilitates the attachment of cancer cells and allows invasion into the vessel wall [22]. Fibrin remodeling is involved in almost all the steps of metastasis, and plays a central role in neovascularization [20, 21]. Within the tumor extracellular matrix, cross-linked fibrin offers a stable platform for endothelial cell migration during angiogenesis and for cancer cell migration during invasion. Even the early stages of tumor development show local fibrin deposition and initiation of angiogenesis [22]. Moreover, fibrin deposits around cancer cells in the circulation helps these cells avoid destruction by natural-killer cells [23]. A comparison of tumor dissemination in control and fibrinogen-deficient mice revealed that the absence of circulating fibrinogen markedly reduced the formation of pulmonary metastases after intravenous injection of cancer cells [24]. Similar results were described in another study of mice tumor model, with fibrinogen-deficiency markedly reducing spontaneous macroscopic metastasis in the lungs and regional lymph nodes. In addition, quantities of pulmonary micrometastases were significantly reduced among fibrinogen-deficient mice after intravenous injection of lung carcinoma cells [25]. Several reports in patients with malignancy have examined the prognostic significance of D-dimer concentrations. Ay et al. prospectively analyzed 1178 cancer patients without VTE over a period of 2 years until VTE or death. Study participants comprised 829 patients (70.4%) with solid tumors, 148 (12.6%) with brain tumors and 201 (17%) with hematological malignancies [26]. Patients were divided into quartiles according to D-dimer concentrations, revealing that high concentrations of D-dimer were associated with significantly poorer survival among patients with any type of malignancy. Other reports have examined the prognostic relevance of D-dimer among patients with primary lung cancer. Taguchi et al. measured D-dimer concentrations in 70 patients with lung carcinoma, finding that low levels of D-dimer were predictive of longer survival [27]. Buccheri et al. demonstrated a correlation between prognosis and D-dimer concentration. For 826 patients with lung carcinoma, median survival times were 154 days for patients with above-normal concentrations of D-dimer, and 308 days for patients with normal concentrations [28].

Altiay et al. investigated the relationship between D-dimer level and prognosis in a study of 78 patients with non-surgically treated primary lung cancer [29]. Zhang et al. investigated 232 patients with resected NSCLC, including 17 patients who developed VTE postoperatively, and confirmed the prognostic relevance of D-dimer concentrations [30]. Although the aim and results of that study showed some overlap with our own, our multivariate survival analysis also adjusted for additional factors strongly associated with prognosis, including CEA, smoking, and SUVmax of the tumor. Moreover, our study population was considered more homogeneous, given the lack of postoperative VTE events.

Uni- and multivariate analyses in this study revealed that increased D-dimer levels were predictive of worsened outcomes. Our findings support previous experimental and clinical findings of enhanced tumor progression and unfavorable outcomes in patients showing up-regulated coagulation and fibrinolytic activities. Such results strongly suggest that interactions between angiogenesis and hemostasis facilitate metastasis in patients with NSCLC. In general, the development of postoperative recurrence is likely due to the establishment of micrometastases or the presence of circulating tumor cells (CTCs) prior to treatment. Such cells appear undetectable with current diagnostic modalities, such as CT and FDG-PET/CT [31]. Given our findings, we speculate that high preoperative levels of D-dimer may be indicative of micrometastasis or CTCs, and thus in turn postoperative recurrence of NSCLC. D-dimer concentrations could therefore offer a predictor of NSCLC recurrence, even though D-dimer is not released by the tumor itself, unlike CEA.

Some limitations must be considered when interpreting the results of this retrospective analysis. The retrospective evaluation of D-dimer levels represents one clear problem. In addition, the study cohort was small, despite being relatively large compared to other such investigations of NSCLC patients.

Given our results and the data from previous basic studies, increased coagulation and fibrinolytic activities appear to be associated with increased risks of tumor progression and metastasis among patients with NSCLC. Concentrations of D-dimer before surgery may also offer a useful marker of recurrence and metastasis in NSCLC patients following radical resection. Furthermore, functional inhibition of fibrinogen and other coagulation factors might represent novel strategies for treating NSCLC. Further investigations are needed to clarify the relationships among circulating coagulation and angiogenic factors in neoplastic tissues.

## Conclusions

The findings of this study suggest that preoperative D-dimer concentration offers an independent predictor of prognosis for completely resected clinical stage I NSCLC, along with age, SUVmax of the primary tumor, and pathological stage. The clinical implications of this finding remain to be determined.

## Abbreviations

CEA: carcinoembryonic antigen
CI: confidence interval
CT: computed tomography
CTCs: circulating tumor cells
D-dimer: plasma D-dimer
DIC: disseminated intravascular coagulation
FDG-PET/CT: integrated 18F–fluorodeoxyglucose positron emission tomography/computed tomography
HR: hazard ratio
NSCLC: non-small cell lung cancer
OS: overall survival
RFS: recurrence-free survival
SUVmax: maximum standardized uptake value
VTE: venous thromboembolism
