# Correction to: Prognostic significance of preoperative plasma D-dimer level in patients with surgically resected clinical stage I non-small cell lung cancer: a retrospective cohort study

**DOI:** 10.1186/s13019-019-0935-6

**Published:** 2019-06-20

**Authors:** Kaoru Kaseda, Keisuke Asakura, Akio Kazama, Yukihiko Ozawa

**Affiliations:** 1 Department of Thoracic Surgery, Sagamihara Kyodo Hospital, 2-8-18 Hashimoto, Midori-ku, Sagamihara, Kanagawa 252-5188 Japan; 2 Department of Pathology, Sagamihara Kyodo Hospital, 2-8-18 Hashimoto, Midori-ku, Sagamihara, Kanagawa 252-5188 Japan; 3 Yuai Clinic, 1-6-2 Kitashinyokohama, Kohoku-Ku, Yokohama, Kanagawa 223-0059 Japan

**Correction to: J Cardiothorac Surg**

**https://doi.org/10.1186/s13019-017-0676-3**

The original version of this article [1] did not cite the following sources [2–5], which were used to write the article.

The authors apologise for any inconvenience that this might have caused.
